# Preparation and Characterisation of Zinc Diethyldithiocarbamate–Cyclodextrin Inclusion Complexes for Potential Lung Cancer Treatment

**DOI:** 10.3390/pharmaceutics16010065

**Published:** 2023-12-31

**Authors:** Ayşe Kaya, Basel Arafat, Havovi Chichger, Ibrahim Tolaymat, Barbara Pierscionek, Mouhamad Khoder, Mohammad Najlah

**Affiliations:** 1 Pharmaceutical Research Group, School of Allied Health, Faculty of Health, Medicine and Social Care, Medical Technology Research Centre, Anglia Ruskin University, Bishops Hall Lane, Chelmsford CM1 1SQ, UKbarbara.pierscionek@aru.ac.uk (B.P.); 2 Biomedical Research Group, School of Life Sciences, Anglia Ruskin University, Cambridge CB1 1PT, UK; 3 Faculty of Health, Science, Social Care and Education, Kingston University London, Kingston upon Thames KT1 2EE, UK

**Keywords:** zinc diethyldithiocarbamate, beta-cyclodextrins, solubility, lung cancer

## Abstract

Zinc diethyldithiocarbamate (Zn (DDC)_2_), a disulfiram metabolite (anti-alcoholism drug), has shown a strong anti-cancer activity in vitro. However, its application was limited by its low aqueous solubility and rapid metabolism. In this study, the solubility enhancement of Zn (DDC)_2_ is investigated by forming inclusion complexes with cyclodextrins. The inclusion complexes were prepared using two different types of beta-cyclodextrins, SBE-CD and HP-CD. Phase solubility diagrams for the resulting solutions were assessed; subsequently, the solutions were freeze-dried for further characterisation studies using DSC, TGA, XRD, and FTIR. The cytotoxic activity of the produced inclusion complexes was evaluated on human lung carcinoma cells using the MTT assay. The solubility of Zn (DDC)_2_ increased significantly upon adding beta-cyclodextrins, reaching approximately 4 mg/mL for 20% *w*/*w* CD solutions. The phase solubility diagram of Zn (DDC)_2_ was of the Ap-type according to the Higuchi and Connors model. Characterisation studies confirmed the inclusion of the amorphous drug in the CD-Zn (DDC)_2_ complexes. The cytotoxicity of Zn (DDC)_2_ was enhanced 10-fold by the inclusion complexes compared to the free drug. Overall, the resulting CD-Zn (DDC)_2_ inclusion complexes have a potential for treatment against lung cancer.

## 1. Introduction

Cancer is a leading cause of death worldwide, characterised by unregulated cellular proliferation that can metastasise to other organs. The World Health Organization (WHO) has reported an estimated 19.3 million cancer cases and over 9.9 million deaths in 2020 [1]. The effectiveness of various cancer therapies is hindered by the severity and frequency of adverse effects, resulting in a higher demand for non-toxic alternatives [2]. The use of metal complexes with anti-cancer activity has had a significant impact on cancer treatments. The discovery of cisplatin cis-[Pt(NH_3_)_2_Cl_2_] in 1845 and the subsequent findings of its biological activity in 1965 stimulated subsequent research on the use of other metal complexes as potential anti-cancer agents [3]. 

Disulfiram (DS) is a clinically approved drug for alcoholism treatment [2]. DS has shown potent anti-cancer activity in a wide range of solid and haematological malignancies [4,5]. The anti-cancer activity of DS is dependent on the availability of divalent cations as copper- and zinc-forming cytotoxic metal complexes [2,6]. Furthermore, it effectively terminates drug-resistant cancer stem cells (CSCs) and reverses chemoresistance [7,8]. DS chelates zinc(II) to produce bis(diethyldithiocarbamate) zinc, a Zn (DDC)_2_ complex. The formation of this complex increases the production of reactive oxygen species (ROS), leading to cancer cell death [9]. Moreover, its cytotoxic activity includes the inhibition of the NF-kB pathway [10]. Additionally, in vitro and in vivo studies showed that DS in the presence of zinc and copper ions suppressed cancer invasion and angiogenesis [11,12]. 

The literature mainly focuses on DS’s ability to bind to copper ions, despite the low concentration of Cu^2+^ in the human body [13]. There is limited research focusing on the relation of DDC with zinc, despite its high affinity for zinc [14]. Moreover, it was found that cancer tissues have higher levels of zinc than normal tissues [15]. Therefore, it is likely that drugs altering zinc levels would be more selective in killing cancerous cells [2]. A case study of patients diagnosed with stage IV ocular melanoma and liver metastases demonstrated the effectiveness of combination therapy using disulfiram and zinc gluconate. This treatment approach successfully induced remission in patients, while causing minimal side effects [16]. These findings have led to further research of this treatment approach through clinical trials where patients with hepatic cancer were treated with disulfiram and copper gluconate [17]. 

Although Zn (DDC)_2_ has shown a superior anti-tumour activity, its application is limited by its poor aqueous solubility in biological fluids and short half-life in the human body, of approximately 4 min [18,19]. Previous research to tackle this issue was based on delivering the water-soluble DDC and a solution of metal salts separately. However, to achieve cytotoxic activity levels, the reaction between DDC and the salt solution must take place inside or adjacently to cancer cells; hence, the delivery of DDC with metal salts as one compound is required [19].

**Figure 1:**
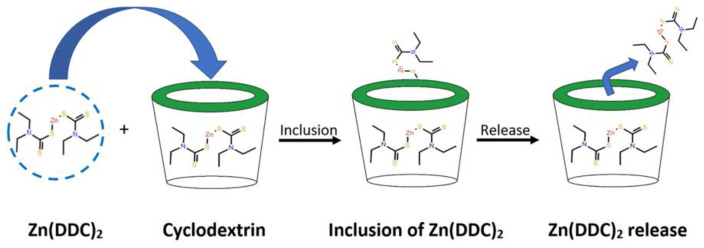
Zn (DDC)_2_ inclusion within the cyclodextrin cavity.

Cyclodextrins (CDs) (Figure 1) are cyclic polysaccharides obtained from the enzymatic degradation of starch, with three native forms αCD, βCD, and γCD consisting of 6, 7, and 8 glucopyranose units, respectively [20]. CDs consist of a lipophilic inner cavity with a hydrophilic exterior surface; due to their truncated cone structure, they possess substantial capabilities to form inclusion complexes in both aqueous solutions and the solid compound state [20,21]. Inclusion complexes are formed by binding forces, such as hydrogen bonds and hydrophobic and van der Waals interactions [22,23]. There are two main factors for forming inclusion complexes: the first is the suitability of CD size for the drug molecule and the second is the optimisation of the thermodynamic interactions between the complex components (CDs, the drug, and the solvent) [22]. CDs have been used as pharmaceutical solubilisers for many decades. The resulting CD–drug inclusion complexes have improved aqueous solubility with increased bioavailability and shelf-life of the host (drug) [24,25]. CDs have the ability to enhance the aqueous solubility of many lipophilic drugs without altering their permeation properties through lipophilic membranes, which make them a more prominent pharmaceutical excipient [23]. Inclusion complexes of cyclodextrins with DS developed to enhance its solubility for the treatment of cataracts and SARS-CoV-2 have been reported [26,27]. The FDA has classified hydroxypropyl beta-cyclodextrin (HP) and sulfobutyl ether beta-cyclodextrin (SBE) as inactive pharmaceutical excipients [25]. CDs are considered safe for parenteral, oral, and pulmonary administration [28,29].

Despite the wide interest in the cytotoxic properties of DS in the presence of metals, there have been a limited number of studies on the cytotoxic activity of Zn (DDC)_2_ for cancer treatment. This is mainly due to its limited solubility and stability. This study aims to enhance the solubility and the stability of Zn (DDC)_2_ by preparing inclusion complexes with CDs. The resulting complexes are characterised by DSC, TGA, FTIR, and X-RD. The cytotoxic activities of the characterised formulations are assessed by MTT assay using lung cancer cell lines. 

## 2. Materials and Methods

### 2.1. Materials

Zinc diethyldithiocarbamate (Zn (DDC)_2_) (molecular weight of 361.99 g/mole and >99.0% purity) was purchased from Tokyo Chemical Industry (TCI) Co., Ltd., Tokyo, Japan. 2-hydroxypropyl-beta-cyclodextrin of USP grade (HP) (molecular weight: 1555 g/mole) and Sulfobutyl ether beta-cyclodextrin sodium (SBE) (molecular weight: 2242.05 g/mole) were bought from Glentham Wiltshire, UK. MD. Acetonitrile laboratory of reagent grade > 99% (molecular weight: 41.05 g/mole) was purchased from Fisher Scientific, Loughborough, UK. Lung cancer cell lines A549 were obtained from ATCC (Teddington, UK). Dulbecco’s modified Eagle’s medium (DMEM), Gibco™ foetal bovine serum (FBS), penicillin streptomycin (Pen-Strep) antibiotic solution, non-essential amino acid solution and L-glutamine (cell culture-tested, 99.0–101.0%), 50 mL centrifuge tubes (sterile), tissue culture flask of 75 cm^2^ (sterile), serological pipettes (sterile), and Gibco™ Trypsin-EDTA (0.25%), were purchased from Fisher Scientific, Loughborough, UK. 3-(4,5-Dimethylthiazol-2-Yl)-2,5-Diphenyltetrazolium Bromide (MTT) and Dimethyl sulfoxide (DMSO) were purchased from Glentham Life Science, Wiltshire, UK. Corning^®^ 96-well TC-treated microplates were obtained from Sigma-Aldrich, Dorset, UK.

### 2.2. Methods

#### 2.2.1. Solubility Study

Solubility studies were carried out using the Higuchi and Connors (1965) method. Aqueous solutions of increasing concentrations of (2-Hydroxypropyl)-β-cyclodextrin (HP) and Sulfobutyl ether β-Cyclodextrin (SBE) cyclodextrins were prepared (1%, 5%, 10%, 15%, and 20% *w*/*w*) using HPLC-grade water. An excess amount of Zn (DDC)_2_ was added to 1 mL of CD solutions. The resulting mixtures were vortexed and then sonicated for two hours at 37 kHz frequency and power of 80%; then, they were agitated for three days at 150 rpm at room temperature using Stuart reciprocating shaker (Cole-parmer, Neots, UK). Afterward, the samples were centrifuged at 13,000 rpm, 22 °C for 10 min (Thermo Scientific Heraeus PIC017, Loughborough, UK). Then, 0.5 mL of the supernatants were transferred to a Spin X centrifuge tube with a cellulose acetate filter (0.45 μm) and centrifuged again using the same method mentioned above. The final filtrates were diluted using HPLC-grade water and analysed using the HPLC method reported by Isa, Rosmahani Che (2009). We used a dionex ultimate 3000 HPLC (Thermo Fisher Scientific UK, Loughborough, UK) with a HyPURITY Advance C8 column (150 mm × 4.6 mm, 5 μm) (Thermo Fisher Scientific UK, Loughborough, UK) and a mobile phase consisting of Methanol: NiCl_2_ (0.01% *v*/*v*) in a ratio of 70:30, at a flow rate of 1 mL/min at 70 °C. The drug concentrations were then calculated from the peak areas, against a calibration curve constructed with the same HPLC method for Zn (DDC)_2_.

#### 2.2.2. Freeze-Drying

Free Zn (DDC)_2_ and its inclusion complexes, SBE-CD and HP-CD, were frozen at −20 °C for two hours and then kept at −80 °C overnight. Using a Lyovapor L-200 freeze-dryer (Buchi Labortechnik, Switzerland), the formulations were freeze-dried for 72 h at a pressure of 0.10 mbar with pressure limit of 0.30 mBar. 

#### 2.2.3. Differential Scanning Calorimetry (DSC) Analysis

DSC analyses of the free Zn (DDC)_2_, SBE-CD, HP-CD, and freeze-dried formulations were performed using a differential scanning calorimetry apparatus (214 Polyma, Netzsch, Selb, Germany). Samples in the range of (3–5 mg) were weighted and crimped-sealed in aluminium led pans using a manual hydraulic press. Both the sample and reference (empty pan) were pierced. The analysis was carried out under a nitrogen gas flow (40 mL/min) at a heating rate of 10 °C /min over a temperature range of 0–300 °C. The resulting thermographs were analysed using the Proteus Analysis software (version 8.0.2). 

#### 2.2.4. Thermogravimetric Analysis (TGA)

TG analyses of the free Zn (DDC)_2_, SBE-CD, HP-CD, physical mixtures, and freeze-dried formulations were carried out using a TG 209 F3 Tarsus model (Netzsch, Germany) to determine the moisture content of the samples. Samples of (3–5) mg were weighted and loaded on a silica crucible suspended and heated from 25 °C to 400 °C with a heating rate of 10 °C/min under a nitrogen gas flow (20 mL/min). The results were analysed using the Proteus Analysis software (version 8.0.2).

#### 2.2.5. X-ray Diffraction Analysis

X-ray powder diffraction patterns of Zn (DDC)_2_, SBE-CD, HP-CD raw materials, inclusion complexes, as well as the physical mixtures were recorded using a Bruker AXS D8 X-ray diffractometer (Karlsruhe, Germany) at a voltage of 20 kV and a 5 mA current. The scanning rate employed was 0.18 s at room temperature, over the range of 11–30° 2θ. The resultant diffraction patterns were analysed using the DIFFRAC plus XRD commander software.

#### 2.2.6. IR Spectroscopy Studies

Infrared spectra were recorded using a Spectrum Nicolet iSF FTIR spectrophotometer (Thermo Scientific, Loughborough, UK). A small quantity of each sample was loaded directly without any treatment on the apparatus with a resolution of 4 cm^−1^ and an average of 65 scans for each measurement. The results are displayed as % transmittance for all samples versus the wavenumber, ranging from 4000 to 650 cm^−1^. The OMNIC 9 software was used to obtain the data. 

#### 2.2.7. Cytotoxicity Studies

The cytotoxic effect of the inclusion complexes HP-Zn (DDC)_2_ and SBE-Zn (DDC)_2_ on the lung cancer cell lines was evaluated in vitro. The viability of the cells was assessed using the MTT assay. A549 cell lines were seeded in 96-well plates at a seeding density of 5 × 10^3^ cells in Dulbecco’s modified Eagle’s medium (DMEM) with 10% FBS, 1 mM sodium pyruvate, 2 mM L-glutamine, 1% penicillin/streptomycin, and 1% non-essential amino acid. The cells were incubated for 24 h at 37 °C with 5% CO_2_ to allow the cells to adhere to the plates. On the next day, the cells were treated with both formulations at the highest concentration of 200 µM. Zn (DDC)_2_ dissolved in DMSO was used as a positive control, while empty CDs were used as a negative control. After 72 h, all wells were treated with a standard 3-(4,5-Dimethylthiazol-2-yl)-2,5-Diphenyltetrazolium bromide (MTT) assay at a concentration of (5 mg/mL). The plates were incubated for 3 h. The MTT solution was aspirated from the wells and replaced with 100 µL of DMSO to dissolve the formazan crystals. The plates were covered with foil and incubated for 5 min. Using the Thermo Scientific MultiSkan FC plate reader, the absorbance was read at 540 nm and data analysis was carried out using Microsoft Excel. The experiment was carried out in triplicate and the IC_50_ values were calculated. 

#### 2.2.8. Statistical Analysis

Statistical significance was measured by Student’s *t*-tests using Excel. All values were expressed as the mean ± standard deviation. Values of *p* < 0.05 were considered as significantly different. 

## 3. Results and Discussion

### 3.1. Solubility Studies

**Figure 2:**
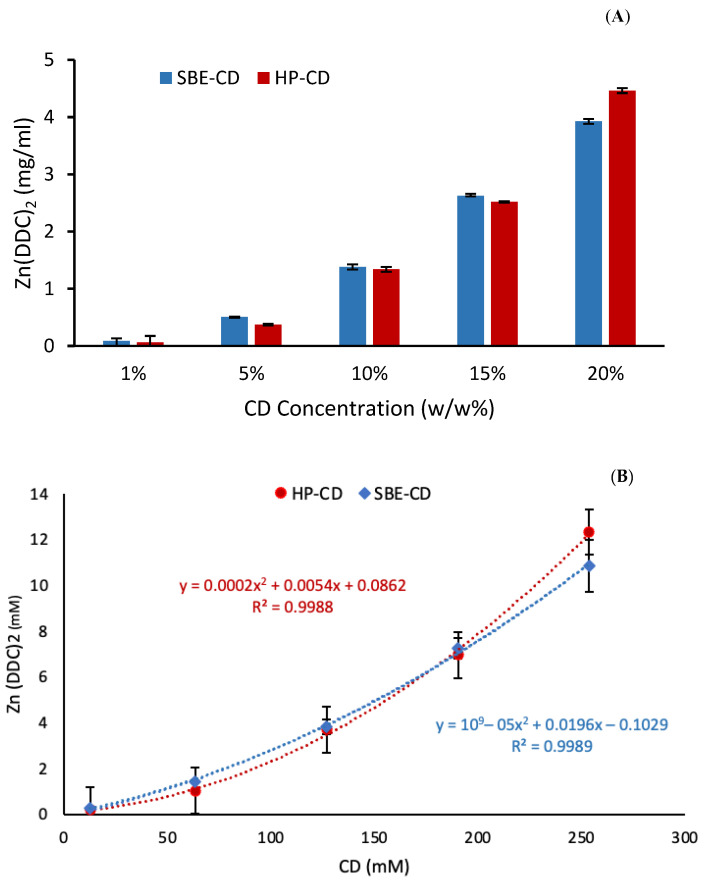
(**A**) Zn (DDC)_2_ solubility (mg/mL) in the CD solutions (*w*/*w*%). (**B**) Phase solubility diagram of Zn (DDC)_2_ in CDs (mM) (mean ± SD, *n* = 3).

The phase solubility diagram for the complex between Zn (DDC)_2_ and both CDs is illustrated in Figure 2. The intrinsic solubility of Zn (DDC)_2_ in water was found to be 0.0006 mg/L, using the method reported by Said Suliman et al. [5]. DMSO was used as a co-solvent due to the limited aqueous solubility of Zn (DDC)_2_ (Figure S1). The water solubility of Zn (DDC)_2_ increased proportionally as a function of the CD concentration up to 20% *w*/*v*. The solubility of Zn (DDC)_2_ displayed a polynomial relationship with the increase in the concentration of both CDs. According to Higuchi and Connors’ phase solubility classification, the obtained phase diagram belonged to the Ap-type (Figure 2B), as the curve deviates positively from linearity, suggesting that using HP-CD or SBE-CD is proportionally more effective at higher concentrations [30]. 

The most common stoichiometry of the inclusion complexes is 1:1, where the complexes are formed by the interaction of one molecule of the drug with one molecule of CD. Generally, the Ap-type phase solubility diagram indicates the formation of a higher complex order, a first order with respect to the guest Zn (DDC)_2_ and a second or higher order with respect to cyclodextrins [31]. However, a deviation from linearity can occur due to solute–solvent interactions, such as hydration, and solute–solute interactions, such as aggregation, association, or both [32,33]. The activity coefficients increase with hydration and decrease with aggregation. For the Zn (DDC)_2_ complexes, the deviation increased as the concentration increased for both CDs. Therefore, cyclodextrins formed complex aggregates that are capable of solubilizing additional amounts of Zn (DDC)_2_ through non-inclusion complex formations or the formation of micelle-like structures [23,34]. Similar findings for Cu (DDC)_2_ were reported by Suliman et al. [5], in which the phase solubility diagram of Cu (DDC)_2_ had a non-linear relationship when forming inclusion complexes with CDs. Upon the inclusion of Zn (DDC)_2_ with CDs, the solubility increased up to 3.92 ± 0.07 and 4.46 ± 0.17 mg/mL for the 20% *w*/*w* SBE-CD and HP-CD complexes, respectively. No significant differences were found between both complexes (*p* > 0.05).

### 3.2. Physiochemical Characterisation of the Freeze-Dried Formulations

#### 3.2.1. Differential Scanning Calorimetry (DSC) Analysis

**Figure 3:**
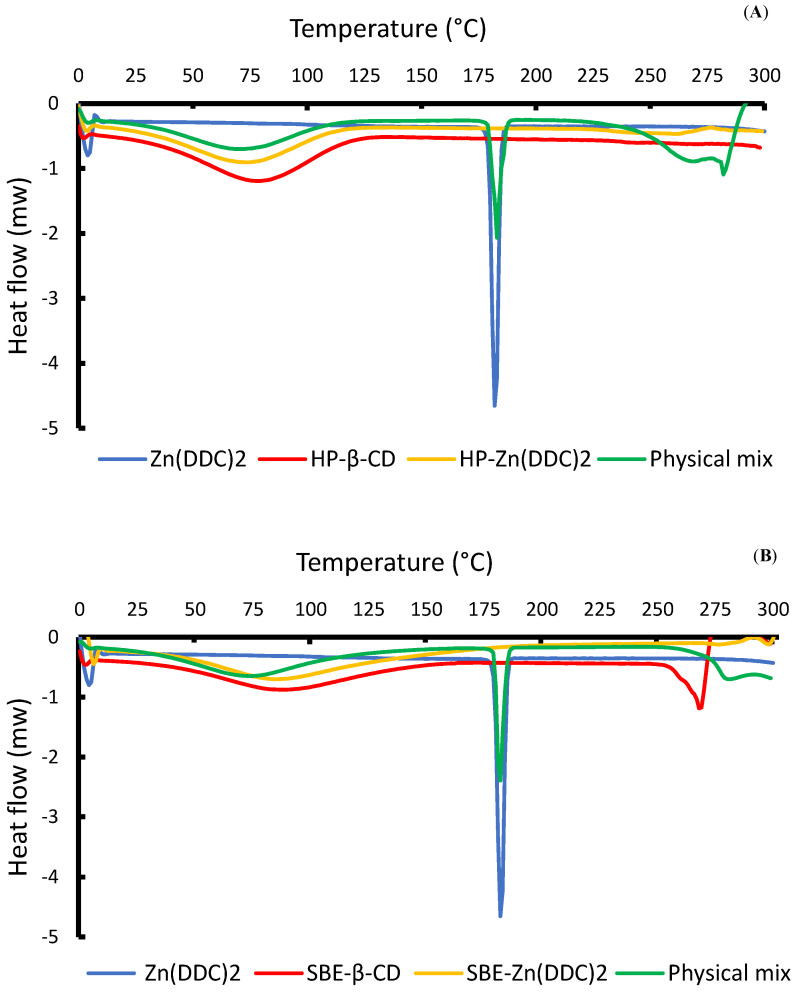
(**A**) DSC thermographs for HP-β-CD, the physical mixtures, and the HP-CD freeze-dried formulations. (**B**) DSC thermographs for Zn (DDC)_2_, SBE-β-CD, the physical mixtures, and the SBE-CD freeze-dried formulations.

DSC was used to verify the formation of the CD-Zn (DDC)_2_ inclusion complexes. When the guest molecules are embedded in CD cavities, their boiling, melting, and sublimation points shift to different temperatures [35]. The DSC thermograms of the free Zn (DDC)_2_, raw CDs, freeze-dried formulations, and physical mixtures are presented in Figure 3. The thermogram of Zn (DDC)_2_ showed a sharp endothermic peak at around 182 °C, which corresponds to the melting point of the drug and indicates its crystalline state, with an enthalpy (ΔH) value of −79.35 J/g. The thermograms of both CDs showed a broad peak at around 85 °C, which was caused by the liberation of water molecules from the CD cavities [36,37]. It can also be observed from Figure 3 that the freeze-dried formulations had similar profiles to those of the raw CDs. Nevertheless, the total absence of the endothermic peak at 182 °C in both formulations suggests that changes that occurred at the molecular level prevented the crystallisation of the guest molecule, thus indicating the amorphous state of Zn (DDC)_2_. This might be an indication that the drug was embedded inside the CD cavities. Moreover, the enthalpy values for HP-CD and SBE-CD were −220.4 J/g and 223.8 J/g, respectively, whereas the enthalpy values of the inclusion complexes HP-Zn (DDC)_2_ and SBE-Zn (DDC)_2_ dropped to −188.7 J/g and −194.6 J/g, respectively. This change in the enthalpy values could be attributed to the formation of the inclusion complexes. Furthermore, the inclusion of the drug inside the CD cavities was confirmed using the physical mixtures of both SBE and HP cyclodextrins with Zn (DDC)_2_. As shown in Figure 3, the distinctive endothermic peak of Zn (DDC)_2_ appeared in the physical mixtures. Hence, this observation confirms the formation of the inclusion complexes.

#### 3.2.2. Thermogravimetric Analysis (TGA)

**Figure 4:**
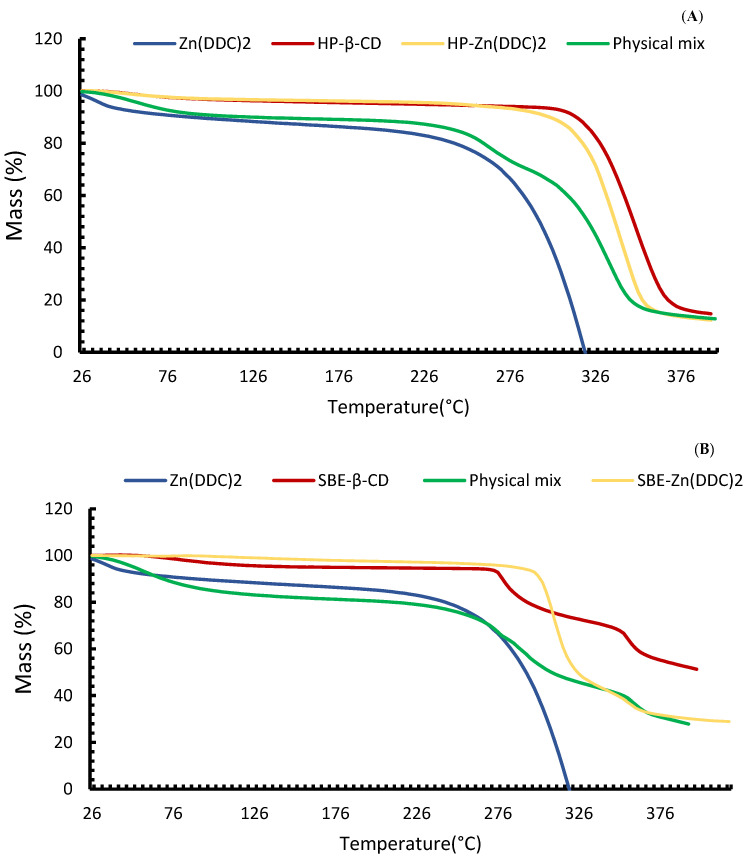
(**A**) TGA thermographs for Zn (DDC)_2_, HP-β-CD, the physical mixtures, and the HP-CD freeze-dried formulations. (**B**) TGA thermographs for Zn (DDC)_2_, SBE-β-CD, the physical mixtures, and the SBE-CD freeze-dried formulations.

TGA is employed to understand the thermostability and crystalline behaviour of particles. TGA was used for the confirmation of the DSC results as changes in the mass provide supportive evidence for the formation of the inclusion complexes, as shown in Figure 4. The TGA thermogram of the pure Zn (DDC)_2_ showed a major weight loss of 80.39% of its mass between 291.2 °C and 324.4 °C. This might be attributed to the cleavage of chemical bonds between Zn^+2^ and DDC, and the subsequent degradation of DDC to diethylamine and carbon disulphide [38]. A similar TGA profile was also reported by Suliman et al. [5] for Cu (DDC)_2_. The water loss of CDs started at around 100 °C followed by a major mass loss starting at 277.1 °C for SBE-CD and 328.8 °C for HP-CD, corresponding to a major thermal degradation. The different TGA profiles for CDs were due to the differences in their chemical structure; more importantly, the TGA thermogram of the inclusion complexes showed a higher thermal degradation for Zn (DDC)_2_. The mass loss started at 320.4 °C and 306.6 °C for the HP and SBE formulations, respectively. Therefore, the thermal stability of Zn (DDC)_2_ shifted to a higher temperature owing to the strong interactions between the drug and CD cavity. The physical mixtures had lower degradation temperatures than those of the inclusion complexes, 303.8 °C and 279.9 °C for HP and SBE, respectively. These observations indicate that CDs provide protection for Zn (DDC)_2_ by forming inclusion complexes [39].

### 3.3. X-ray Diffraction Analysis

**Figure 5:**
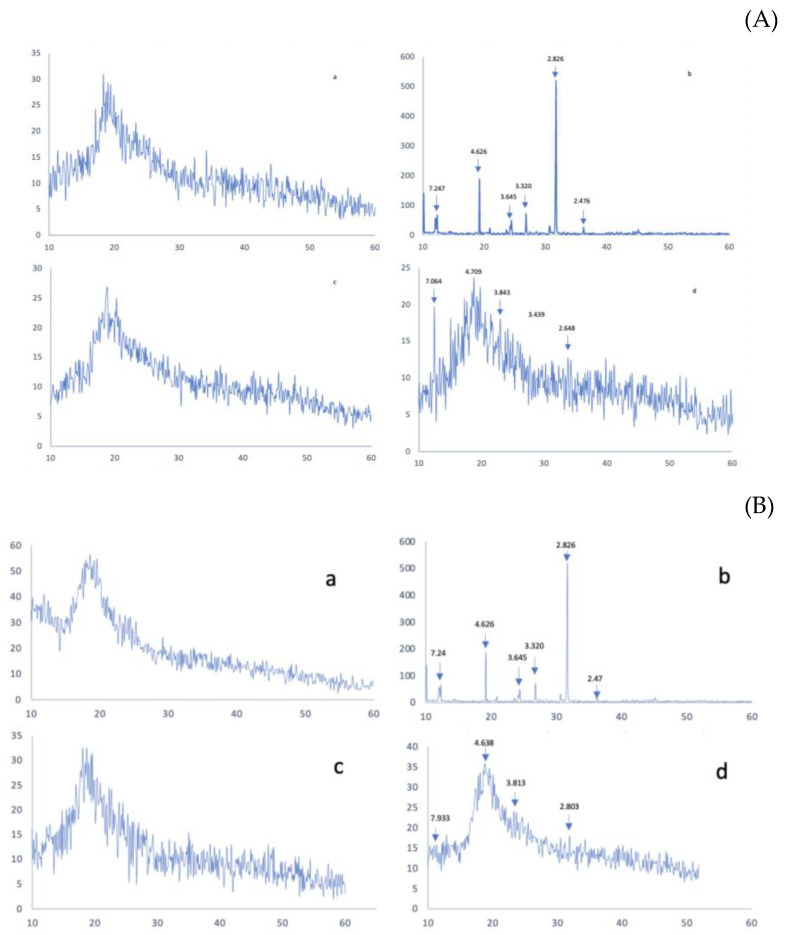
(**A**) X-ray powder diffraction (XRD) patterns of (a) SBE-CD, (b) Zn (DDC)2, (c) SBE-Zn (DDC)2, and (d) the physical mixtures. (**B**) X-ray powder diffraction (XRD) patterns of (a) HP-CD, (b) Zn (DDC)2, (c) HP-Zn (DDC)2, and (d) the physical mixtures.

Powder-ray diffractometry is another technique for evaluating complex decomposition and complex formation. The XRD pattern of the sample is usually determined as a function of the scattering angle [2]. The diffraction patterns of the pure Zn (DDC)_2_, both cyclodextrins, and physical mixtures corresponding to the solid inclusion complexes with CDs are presented in Figure 5. The X-ray diffractogram of Zn (DDC)_2_ presented a sharp diffraction peak at 2θ of 4.6°, 3.6°, 3.3°, 2.9°, and 2.8°, indicating the crystalline state of the drug. The two CDs showed a broad peak in the range of 15–20 (2θ), which is the characteristic peak of B-cyclodextrins, as described in the literature [40,41]. The sharpening of peaks and the disappearance or appearance of peaks in the X-ray diffraction patterns indicates complex formation [42]. Accordingly, the total amorphisation of the drug in both CD formulations suggests that the drug is embedded within the CD cavities. This observation was further confirmed using the physical mixtures, in which the drug peaks were still detectable at 2θ of 4.7°, 3.8°, and 2.8°. However, the intensity of the peaks in the physical mixtures was reduced in comparison to that of the pure drug, which might be due to the lower drug content in the physical mixtures.

### 3.4. IR Spectroscopy Studies

**Figure 6:**
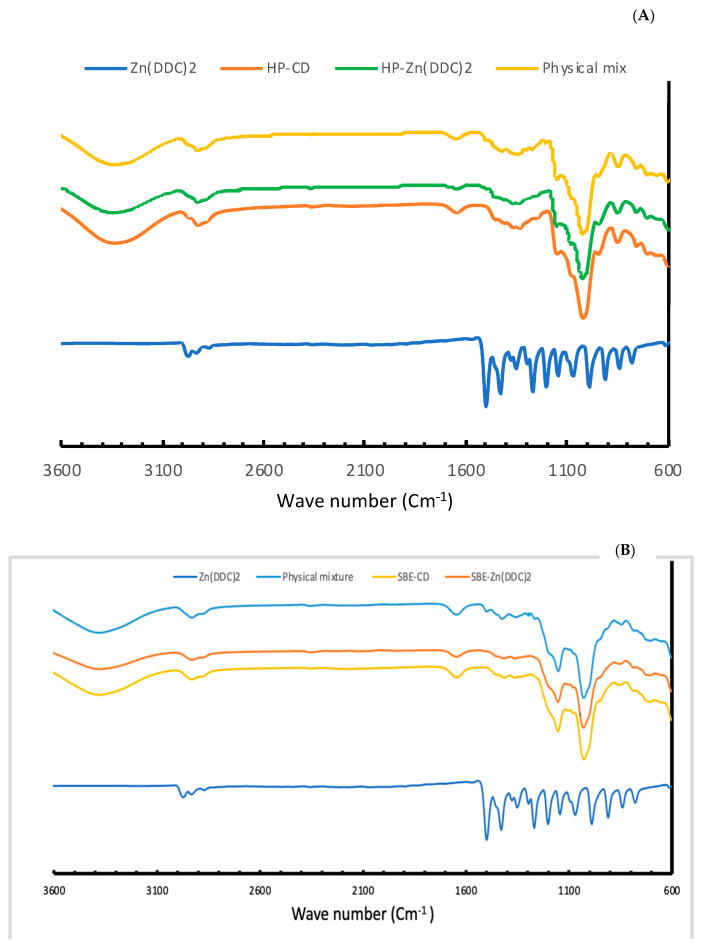
(**A**) IR spectra of Zn (DDC)_2_, HP-β-CD, the freeze-dried formulations, and the physical mixtures. (**B**) FTIR spectra of Zn (DDC)_2_, SBE-β-CD, the freeze-dried formulations, and the physical mixtures.

The overlaid IR spectra of the raw Zn (DDC)_2_, CDs, the freeze-dried formulations of SBE-CD and HP-CD, and the physical mixtures are shown in Figure 6. The IR spectra of both CDs were almost identical with some variations. The broad absorption bands in the range of 3600–3200 cm^−1^ correspond to the OH groups stretching, which is a characteristic peak for cyclodextrins. An intense peak due to C-H stretching can be seen at 2900 cm^−1^. At 1600 cm^−1^, the deformation band of water H-OH in cyclodextrins can be observed. Between 1100 and 1200 cm^−1^, C-H vibrations can be seen. The absorptions at 1300 to 600 cm^−1^ correspond to the skeletal vibrations of C-C and C-O-C. As it is shown in Figure 4, in the IR spectra of Zn (DDC)_2_, there is a clear aliphatic C-H stretch at 2900 cm^−1^. Also, the strong absorption band around 1500 cm^−1^ indicates a C-N partial double bond. The IR spectra of the freeze-dried formulations were similar to the ones of the raw CDs. More importantly, the vibration bands in the Zn (DDC)_2_ spectra did not appear in the IR spectra of the freeze-dried formulations. Wang et al. have also reported similar results for CD-Zn (DDC)_2_ inclusion complexes. This might indicate the inclusion of the drug inside the CD cavities. On the other hand, the vibration bands corresponding to Zn (DDC)_2_ did not appear in the IR spectra of the physical mixtures. Therefore, the disappearance of the drug vibrations in the formulations might be due to the content of the drug in the formulations being lower than the limit of detection of the FTIR. A similar phenomenon was reported by Suliman et al. for Cu (DDC)_2_ inclusion complexes [5]. Finally, further investigation are needed to obtain a clear explanation of the interaction between CD and Zn (DDC)_2_ in the 1H NMR spectrum, for example.

### 3.5. Cytotoxicity Studies

**Figure 7:**
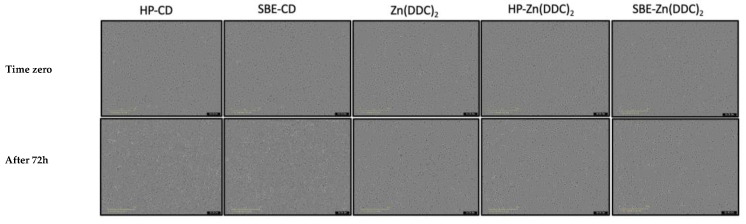
The morphology (×100 magnification) of lung cancer cells at time zero and after 72 h of exposure to Zn (DDC)_2_ (50 µM) and its cyclodextrin complexes (equivalent to 50 µM).

**Figure 8:**
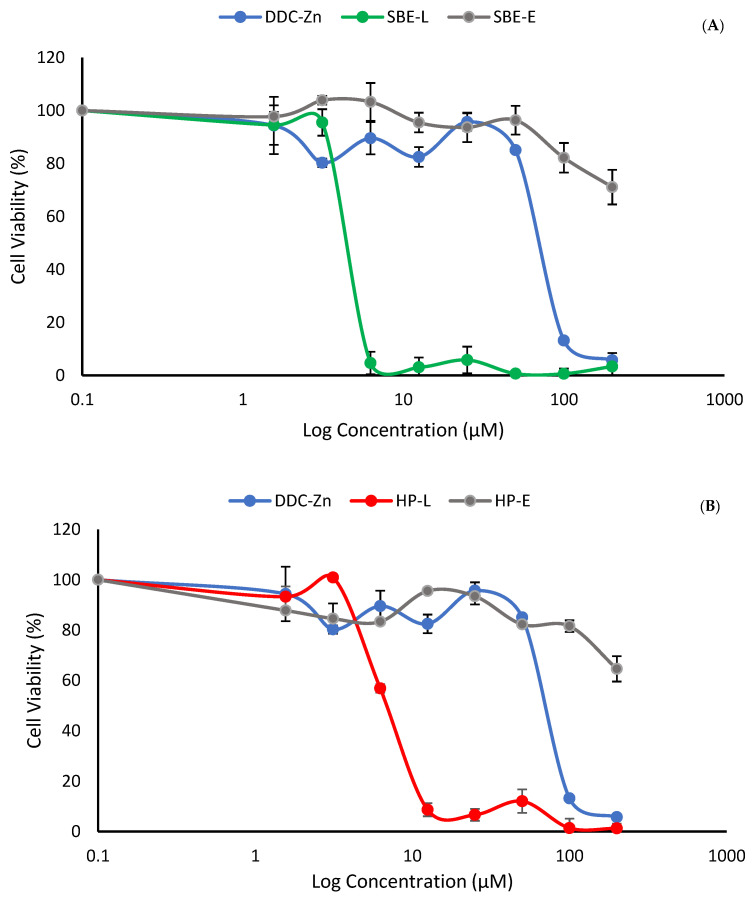
(**A**) Survival curves for A549 lung cancer cell lines after 72 h exposure to the SBE-Zn (DDC)_2_ complex and the free drug. (**B**) Survival curves for A549 lung cancer cell lines after 72 h exposure to the HP-Zn (DDC)_2_ complex and the free drug (mean ± SD, *n* = 3).

The effect of free Zn (DDC)_2_ and its inclusion complexes HP-Zn (DDC)_2_ and SBE-Zn (DDC)_2_ on the viability of lung cancer cell lines (A549) was determined by the MTT assay. Figure 6 shows the morphological changes of the cells after the exposure to IC_50_ concentrations of the free drug and cyclodextrin complexes. It can be seen clearly in Figure 7 and Figure 8 that the cytotoxicity levels of HP-Zn (DDC)_2_ and SBE-Zn (DDC)_2_ on A549 cells were stronger than that of the free Zn (DDC)_2_. In the absence of Zn (DDC)_2_, HP-CD and SBE-CD did not show any cytotoxicity on A549 cells. It was previously reported that cyclodextrins have no cytotoxic effect [24]. Table 1 shows the IC_50_ values of the free drug and the inclusion complexes. The IC_50_ values of HP-Zn (DDC)_2_ and SBE-Zn (DDC)_2_ were 6.8 ± 0.57 µM and 4.9 ± 0.34 µM, respectively. No significant difference between the IC_50_ values of both formulations was observed (*p* > 0.05). The IC_50_ values of the inclusion complexes were almost 10-fold lower than that of free Zn (DDC)_2_ (IC_50_ = 54.6 ± 1.59). Therefore, the anti-cancer activity of Zn (DDC)_2_ was enhanced upon its complexation with CD. This observation suggests the enhanced solubility and the efficient endocytic uptake of entrapped Zn (DDC)_2_. These findings agree with those of other studies, where using cyclodextrin inclusion complexes improved the cytotoxicity of the guest molecule [40,41,42,43].

## 4. Conclusions

This study highlighted that the inclusion of Zn (DDC)_2_ into CD complexes may overcome the poor solubility issues that hinder its development as a cytotoxic agent. The solubility of Zn (DDC)_2_ was significantly increased using a complexation method with cyclodextrins. The formation of CD-Zn (DDC)_2_ inclusion complexes was investigated using the thermogravimetric analyses TGA and DSC alongside IR and X-RD. The MTT cytotoxicity study elucidated an improvement in the anti-cancer activity of the drug upon the formation of the inclusion complexes in compared to the free drug. Hence, the resulting complexes have a high potential for further anti-cancer application.

## Figures and Tables

**Table 1:** The IC_50_ values of Zn (DDC)_2_ and its formulations on lung cancer cell lines, *n* = 3 ± SD.

Formulation	IC_50_ µM
Zn (DDC)_2_	54.63 ± 1.59
HP-Zn (DDC)_2_	6.81 ± 0.57
SBE-Zn (DDC)_2_	4.95 ± 0.34

## Data Availability

The data presented in this study are complemented by the Supplementary Materials. Enquiries may be made to the corresponding author.

## Supplementary Materials

The following supporting information can be downloaded at: https://www.mdpi.com/article/10.3390/pharmaceutics16010065/s1. Figure S1: Zn (DDC)_2_ solubility in DMSO/water mixtures against DMSO concentrations *v*/*v*%; Figure S2: (A) X-ray powder diffraction (X-RD) pattern of (a) SBE-CD, (b) Zn (DDC)_2_, (c) SBE-Zn (DDC)_2_, and (d) the physical mixtures. (B) X-ray powder diffraction (X-RD) pattern of (a) SBE-CD, (b) Zn (DDC)_2_, (c) SBE-Zn (DDC)_2_, and the (d) physical mixtures; Figure S3: Stability study of the freshly prepared SBE and HP inclusion complexes with Zn (DDC)_2_.
