# Supplementary material for: Preparation and Characterisation of Zinc Diethyldithiocarbamate–Cyclodextrin Inclusion Complexes for Potential Lung Cancer Treatment
Source: Pharmaceutics. 2023 Dec 31;16(1):65. doi: 10.3390/pharmaceutics16010065 (PMC10819758; doi:10.3390/pharmaceutics16010065)
Supplement: Supplementary file 1 [file pharmaceutics-16-00065-s001.zip › pharmaceutics-2772713-supplementary.pdf]

pharmaceutics

PREPARATION AND CHARACTERISATION OF DIETHYLDITHIOCARBAMATE ZINC CYCLODEXTRIN INCLUSION COMPLEXES FOR POTENTIAL LUNG CANCER TREATMENT

Ayşe Kaya[1], Basel Arafat[1], Havovi Chichger [2] , Barbara Pierscionek[1], Mouhamad Khoder[3], and Mohammad Najlah[1,*]

1. Intrinsic solubility prediction of $Zn(DDC)_2$ in water

1.1 Methods

DMSO was used as co-solvent to determine the intrinsic aqueous solubility of $Zn(DDC)_2$. Following the same method for solubility determination of Zn $(DDC)_2$ with cyclodextrin but with the absence of cyclodextrin. Zn $(DDC)_2$ was added to DMSO/water mixture using different ratios (70, 75, 80, 85 and 95 % v/v) of DMSO. The resulting solutions were sonicated for two hours then agitated for 3 days using Stuart shaker, 150 rpm at room temperature to reach equilibrium. Afterwards, the solutions were centrifuged for 10 min at 13,000 rpm. The supernatants were transferred into Spin X centrifuge tube with 0.45um filter and centrifuged again under the same conditions. Drug concentrations were determined using the reported HPLC method.

1.2 Results

The solubility of Zn $(DDC)_2$ in DMSO/water mixtures is exponential with DMSO concentration figure (S1). Similar findings were reported for the intrinsic solubility of Cu $(DDC)_2$ in DMSO (Suliman et al., 2021). The intrinsic solubility can be determined from the value of the intercept in the exponential equation below. Therefore, the intrinsic solubility of Zn $(DDC)_2$ in water is 0.0006 mg/L.

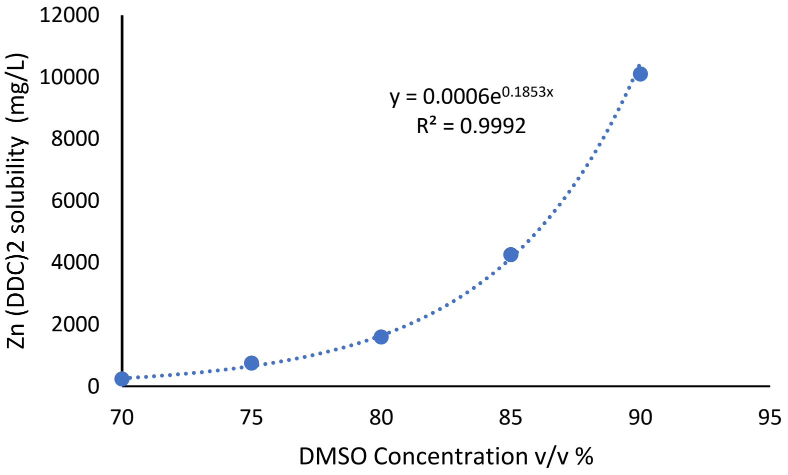

Figure S1. Zn $(DDC)_2$ solubility in DMSO/water mixtures against DMSO concentrations v/v%

2.Drug Precipitation Study

2.1 Method

The drug content of freshly prepared formulations was assessed over three months. Samples were stored at room temperature and at intervals of a week, drug concentrations were tested by HPLC method explained earlier.

2.2 Results

One of the challenges for the preparation of any formulation encapsulating Zn $(DDC)_2$ is its rapid precipitation in the form of crystals. The stability of freshly prepared formulations for both HP-CD and SBE-CD was performed for six months, to confirm that there was no crystallisation, precipitation, or degradation of Zn $(DDC)_2$. Figure (S2) illustrates that both solutions were stable with no significant change in Zn $(DDC)_2$ concentration ($p > 0.05$). These findings correlate with cyclodextrins properties of enhancing the chemical stability of the drugs and improving its shelf-life (Popielec and Loftsson, 2017).

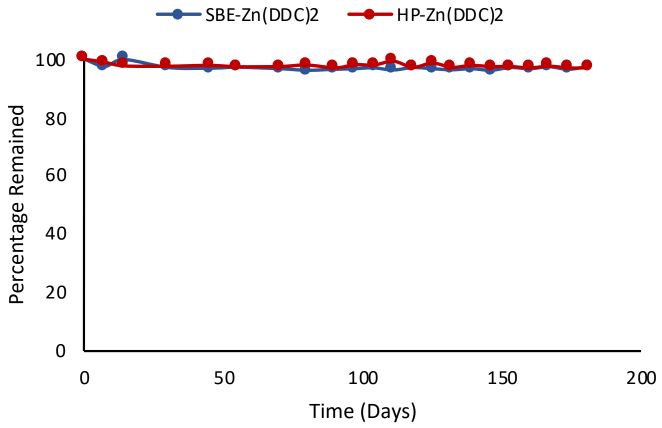

Figure S2. Stability study for freshly prepared SBE and HP Inclusion complexes with Zn $(DDC)_2$

[1] Denadai, M. L.; Santoro, M. M.; Lopes, M. T. P.; Chenna, A.; De Sousa, F. B.; Avelar, G. M.; Gomes, M. R. T. ´ U.; Guzman, F.; Salas, C. E.; Sinisterra, R. D. A Supramolecular Complex between Proteinases and β-Cyclodextrin That Preserves Enzymatic Activity; 2006; Vol. 20.

[2] Wang, X.; Parvathaneni, V.; Shukla, S. K.; Kanabar, D. D.; Muth, A.; Gupta, V. Cyclodextrin Complexation for Enhanced Stability and Non-Invasive Pulmonary Delivery of Resveratrol—Applications in Non-Small Cell Lung Cancer Treatment. AAPS PharmSciTech, **2020**, 21 (5). https://doi.org/10.1208/s12249-020-01724-x.

[3] Popielec, A.; Loftsson, T. Effects of Cyclodextrins on the Chemical Stability of Drugs. Int J Pharm, **2017**, 531 (2), 532–542. https://doi.org/10.1016/j.ijpharm.2017.06.009.
